# Human Neutrophil Antigen Genotype and Allele Frequencies in Iranian Blood Donors

**DOI:** 10.1155/2022/4387555

**Published:** 2022-02-07

**Authors:** Behnaz Esmaeili, Behnaz Bayat, Atefe Alirezaee, Mona Delkhah, Mohammad Reza Mehdizadeh, Zahra Pourpak

**Affiliations:** ^1^Immunology, Asthma and Allergy Research Institute, Tehran University of Medical Sciences, Tehran, Iran; ^2^Institute for Clinical Immunology and Transfusion Medicine, Justus Liebig University, Giessen, Germany; ^3^Flow Cytometry Department, Children Medical Center, Tehran University of Medical Sciences, Tehran, Iran; ^4^Blood Transfusion Organization Center, Tehran, Iran

## Abstract

**Objective:**

Human neutrophil antigens (HNAs) can be targeted by HNA-allo antibodies and cause a variety of clinical conditions such as transfusion-related acute lung injury (TRALI) and neonatal alloimmune neutropenia (NAIN). The current study is aimed at identifying the genotype and allele frequencies of HNAs in Iranian blood donors.

**Methods:**

A total of 150 blood samples were obtained from healthy blood donors. HNA-1, HNA-3, HNA-4, and HNA-5 were genotyped, using the polymerase chain reaction sequence-specific primer (PCR-SSP) technique. The expression of the HNA-2 antigen on the neutrophil surface was evaluated by flow cytometry.

**Results:**

The allele frequencies of FCGR3B∗1 (encoding HNA-1a), FCGR3B∗2 (encoding HNA-1b), and FCGR3B∗3 (encoding HNA-1c) were 0.34, 0.63, and 0.03, respectively. For HNA-3, the allele frequencies for SLC44A2∗1 (encoding HNA-3a) and SLC44A2∗2 (encoding HNA-3b) were 0.63 and 0.37, respectively. The frequencies of ITGAM∗1 (encoding HNA-4a) and ITGAM∗2 (encoding HNA-4b) alleles were 0.85 and 0.15, respectively. Furthermore, the frequencies of ITGAL∗1 (encoding HNA-5a) and ITGAL∗2 (encoding HNA-5b) alleles were 0.72 and 0.28, respectively. In the studied population, HNA-2 antigen was present on the neutrophil surface in 97.3% of the individuals, while no detectable HNA-2 expression was observed in 2.7% of the individuals. However, no significant difference in HNA-2 expression between different age groups was found.

**Conclusion:**

The present study provides the first report of the HNA allele and genotype frequencies among the Iranian population. All HNAs (HNA-1 to HNA-5) were typed using the PCR-SSP and flow cytometer. In the current cohort study, the determined HNA allele frequencies were similar to the previous reports from British, German, and Danish populations. Considering the presence of different Iranian ethnic groups, further studies with a larger sample size are needed to draw a total picture for HNA allele frequencies.

## 1. Introduction

Human neutrophil antigens (HNAs) are glycoproteins expressed on the human neutrophils surface [1]. On human neutrophils, there are five antigenic systems (from HNA-1 to HNA-5) that consist of fourteen alleles located on the human neutrophil Fc gamma-receptor IIIb (Fc*Y*RIIIb) (CD16), CD177, choline transporter-like protein 2 (CTL2), Mac1 (CD11b/CD18), and LFA1 (CD11a/CD18), respectively [2]. Among HNAs, HNA-1 and HNA-2 have been identified only on the neutrophil surface [3]. However, HNA-3, HNA-4, and HNA-5 follow a broad expression pattern on other blood cells [2]. Based on CD177 expression, in HNA-2-positive individuals, one or two neutrophil subsets can be observed. Nonetheless, in some individuals (3-5% of normal population), no CD177 expression on the neutrophil surface is detectable [4]. In the case of atypical expression of HNA-2, one negative and two positive subpopulations of neutrophils are detected [5].

HNA incompatibility in the case of pregnancy or transfusion/transplantation leads to alloimmunization and the consequent production of alloantibodies. The binding of these alloantibodies on target HNAs may lead to a variety of clinical conditions such as transfusion-related acute lung injury (TRALI) and neonatal alloimmune neutropenia (NAIN) [6]. Autoantibodies directed against HNAs are known to involve in the mechanism of autoimmune neutropenia [6].

HNA allele frequencies have been extensively studied among different populations such as Japanese [7], Chinese [8], Hong Kong [9], Thai [10], Turkish, German [11], and Africa American [12], African Blacks [13], northern Germany and Uganda [14], and Danish and Zambian [15].

Previous studies from Iranian populations have investigated the frequencies of HNA-3 and HNA-5 alleles in Iranian blood donors [16, 17]. However, there was no previous report related to the frequencies of all HNAs for Iranian populations. Given that Iran contains multiple ethnic groups, evaluation of the HNA allele frequencies provides useful information to estimate the HNA alloimmunization rate and help to design prevention strategies for the consequences of HNA alloimmunization such as NAIN, TRALI, and graft rejection. The aim of this study was to evaluate HNA allele and genotype frequencies among the Iranian population.

## 2. Materials and Methods

### 2.1. Study Population

Peripheral blood samples (In EDTA-containing vacutainer tubes) from 150 healthy donors referred to the Vesal Blood Transfusion Center, Tehran, Iran (18 females and 132 males, aged 18-61 years), were collected. This study was approved by the Ethics Committee of Tehran University of Medical Sciences (IR.TUMS.IAARI.REC.1398.019). All subjects were interviewed and signed a consent form to participate in the study, and then, 5 mL whole blood was taken from each participant. Additionally, demographic data of subjects including sex, age, and ethnicity were collected. The data of questionnaires indicating the presence of six Iranian ethnic groups in the studied cohort has been summarized in Table 1.

## 3. DNA Extraction and Genotyping for FCGR3B (FCGR3B∗01, FCGR3B∗02, FCGR3B∗03), SLC44A2, ITGAM, and ITGAL Alleles

DNA was extracted from whole blood, using a DNA extraction kit (Exgene Cell SV mini, Gene All, Cat. no.: 106-101, Seoul, South Korea) according to the manufacturer's instructions. Allele and genotype frequencies for HNA-1, HNA-3, HNA-4, and HNA-5 were analyzed by the polymerase chain reaction sequence-specific primer (PCR-SSP) method. The primer sequences used for HNA genotyping are listed in Table 2 [18] (the primer sequences for HNA3a, HNA3b, HNA4a, and HNA4b were obtained from the ISBT reference granulocyte laboratory in Giessen). Briefly, 1 *μ*L DNA (100 ng/*μ*L) was amplified in a total volume of 12 *μ*L. The reaction mix was 6 *μ*L Taq 2x Master Mix (Taq DNA Polymerase, Ampliqon, Cat. no: A180301, Denmark) and 1 *μ*L specific primers (10 *μ*M). As an internal control for PCR reaction, the human growth hormone gene (HGH) (2 *μ*M) was amplified. The PCR amplification conditions for FCGR3B∗1 (encoding HNA-1a), FCGR3B∗2 (encoding HNA1b), SLC44A2∗1 (encoding HNA-3a) and SLC44A2∗2 (encoding HNA-3b), ITGAM∗1 (encoding HNA-4a) and ITGAM∗2 (encoding HNA-4b) *alleles* were same. The PCR amplification was performed using a thermal cycler (ASTEC, Japan) as follows: 3 min initial denaturation at 95°C, followed by 35 cycles of 30 s denaturation at 95°C, 35 s at annealing temperature (different for each set of primers), and 30 s at 72°C and one cycle of 5 min at 72°C for the final extension.

**Figure 1:**
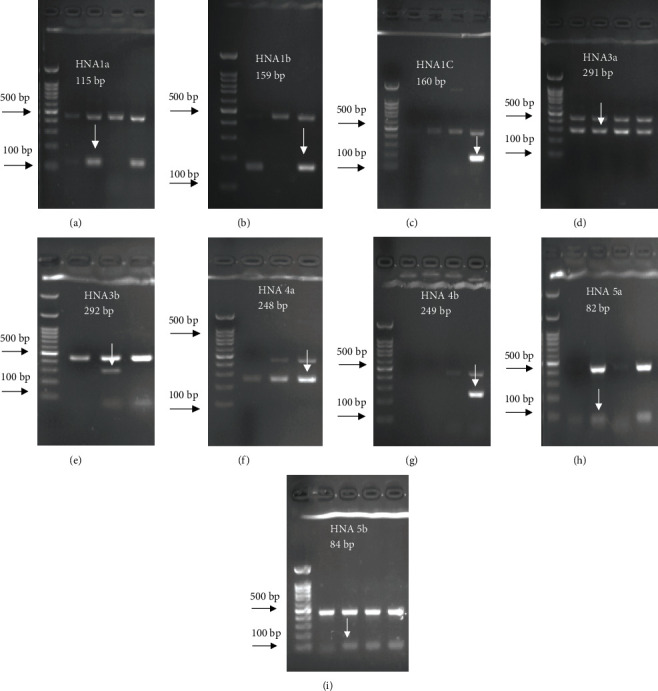
HNA allele-specific amplicons separated by agarose gel electrophoresis. Gel electrophoresis images are related to *FCGR3B* (*FCGR3B*∗*01*, *FCGR3B*∗*02*, *FCGR3B*∗*03*), *SLC44A2*, *ITGAM*, and *ITGAL alleles* on 1.5% agarose. The presence of each allele in the DNA samples was confirmed by the amplification of the related gene fragments using specific primers by polymerase chain reaction sequence-specific primer (PCR-SSP). (a–c) Individuals with *FCGR3B*∗*01* (*encoding* HNA-1a), *FCGR3B*∗*02* (*encoding* HNA-1b), and *FCGR3B*∗*03* (*encoding* HNA-1c) positive alleles, respectively. (d, e) Individuals with positive SLC44A2∗1 (encoding HNA-3a) or SLC44A2∗2 (encoding HNA-3b) alleles, respectively. (f, g) Individuals with positive ITGAM∗1 (encoding HNA-4a) or ITGAM∗2 (encoding HNA-4b) alleles, respectively. (h, i) Individuals with positive ITGAL∗1 (encoding HNA-5a) or ITGAL∗2 (encoding HNA-5b) alleles, respectively. Sequence-specific products are shown by arrows. Human growth hormone (HGH) (495 bp) was used as an internal positive control for all PCR-SSP analyses. A DNA ladder (100–1000 bp) has been run in the first lane of the gels (on the left side) (Biofact, SM342-500, Korea).

For FCGR3B∗3 (encoding HNA-1c) and ITGAL∗1 (encoding HNA-5a) and ITGAL∗2 (encoding HNA-5b) *alleles*, a touchdown PCR amplification was performed using BioRad MyCycler thermal cycler with the following reaction cycles: The initial denaturation step was 2 min at 95°C. At the first stage, the temperature was gradually decreased by 0.5°C following each cycle. The second stage consisted of 20 amplification cycles. In both stages, denaturation and elongation steps were held constant for 30 and 10 seconds at 95°C and 72°C, respectively. The final elongation step was planned at 72°C for 5 min after stage 2. Then, 5 *μ*L of the PCR products was run on 1.5% agarose gel to confirm the correct size of the PCR products (Figure 1). To check the correct target gene amplification, sequencing of the PCR products was performed (data not shown).

## 4. Analysis of the CD177 Expression on the Neutrophil Surface

Flow cytometry was applied to evaluate the expression of the HNA-2 antigen on the neutrophil surface of donors. For this purpose, 100 *μ*L whole blood was incubated with FITC-conjugated antibody specific for CD177 (clone MEM-166; Biolegend, Cat. no.: 315804) for 20 min at room temperature. Following the incubation period, the red blood cells (RBCs) were lysed by applying a lysis reagent (CMGRBL, 10x, Iran). The suspended cells were washed with phosphate-buffered saline (PBS) and immediately examined by flow cytometry. Mouse IgG was used as isotype control (Biolegend). For each sample, approximately 20,000 events were acquired on a BD FACSCanto ™ II flow cytometer. Flow cytometry data was analyzed in the Flowjo software (version 7.6).

## 5. Statistical Analysis

**Figure 2:**
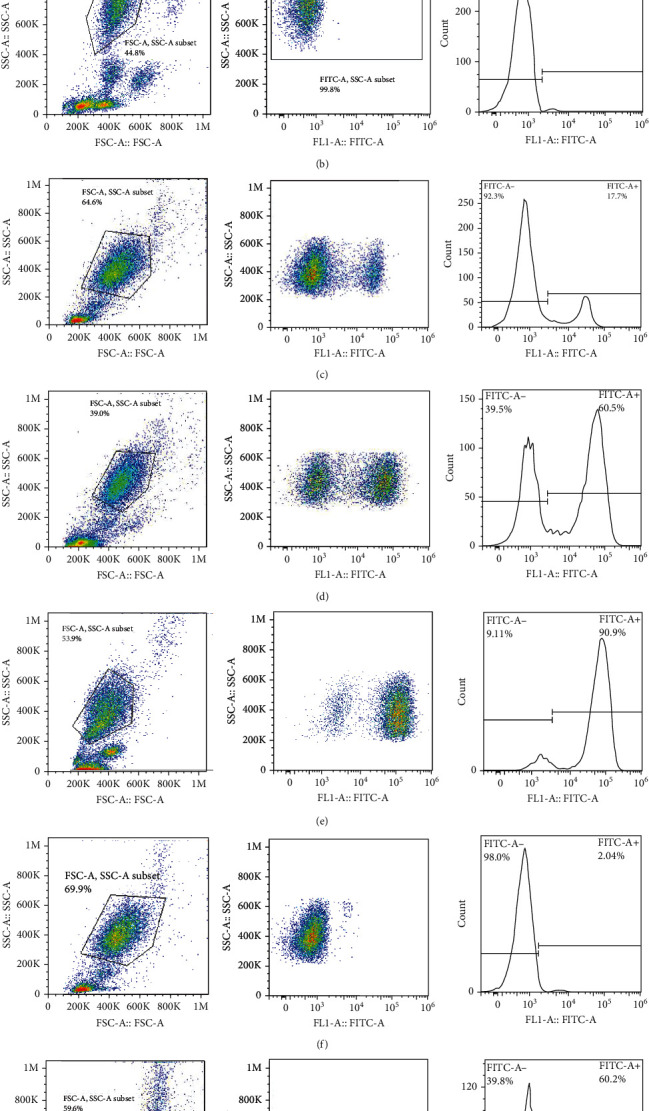
Flow cytometry analysis of HNA-2 expression on the neutrophils. The neutrophils were stained in whole blood with FITC conjugated anti-CD177 antibody. (a, b) Unstained and isotype controls were used to set up the flow cytometer and analyze the results. Based on HNA-2 expression, two distinct populations were identified. Among donors, the expression of the HNA-2 antigens was varied. For analysis, individuals were divided into four categories: (c) HNA-2 expression between 5% and 50%, (d) HNA-2 expression between 50% and 60%, (e) HNA-2 expression more than 60%, and (f) with the HNA-2 expression less than 5% (negative). (g) Individuals with two positive populations were analyzed as atypical expression of the HNA-2 antigen. In the histogram plots, CD177 positive and negative neutrophils were identified using bifurcated gates.

The frequencies of HNA alleles were obtained by the numbers of observed genotypes. The Chi-square test was used to evaluate any deviation of the observed numbers of genotypes from the expected numbers on the Hardy–Weinberg equilibrium and to compare the difference in HNA genotype frequencies among different populations. For HNA-2, the subjects were grouped based on the antigen expression into four categories, according to a previous report [19]: group 1, strong expression (more than 60%); group 2, intermediate expression (50%-60%); group 3, weak expression (5% to 50%); and group 4, negative reaction (<5%) (Figure 2). ANOVA was used to compare the expression of HNA-2 between different age groups. *p* values less than 0.05 were considered significant.

## 6. Results

### 6.1. HNA Allele Frequencies

A totally of 150 samples were analyzed for HNA-1, HNA-3, HNA-4, and HNA-5 allele frequencies and HNA-2 expression. The allele frequencies related to the HNA-1, HNA-3, HNA-4, and HNA-5 are depicted in Table 3. For HNA-1, FCGR3B∗2 showed the highest allele frequency (0.63). FCGR3B∗1 and FCGR3B∗3 frequencies were 0.34 and 0.03, respectively. Regarding HNA-3, HNA4, and HNA-5 antigens, the allele a was the most common. The frequency of SLC44A2∗1 and SLC44A2∗2 alleles was 0.63 and 0.37, respectively. For ITGAM∗1 and ITGAM∗2 alleles, a frequency of 0.85 and 0.15 was obtained, while the frequency for ITGAL∗1 and ITGAL∗2 alleles was 0.72 and 0.28, respectively.

Genotyping data for HNA-1, HNA-3, HNA-4, and HNA-5 as well as HWE analysis are shown in Table 4. Among studied subjects, the HNA-1-related gene was deleted only in one donor (0.7%). However, no donor with all three FCGR3B alleles was identified. Chi-square (*X*^2^) test was used to evaluate the goodness of fit of the study population to Hardy-Weinberg equilibrium. For this purpose, the genotype frequencies were calculated based on allele frequencies, and then, the significant difference between observed and expected frequencies was evaluated using the *X*^2^ test. The respective *p* values obtained after comparing the difference between observed and expected frequencies for HNA-1, HNA-3, HNA-4, and HNA-5 were 0.125, 0.799, 0.740, and 0.915, respectively. According to the obtained *p* values (all >0.05), there was no significant deviation for any of genotypes tested in the study population from Hardy-Weinberg equilibrium (Table 4).

## 7. HNA-2 Expression on the Neutrophil Surfaces from Iranian Blood Donors

The expression of HNA-2 on the neutrophil surface was evaluated by flow cytometry, using fluorescence-labeled anti-CD177 mab. Based on HNA-2 expression, donors were categorized into four groups (negative, low, middle, and high expressing) (Figures 2(c)–2(f)). In the studied cohort, HNA-2 was detected in 146 donors (97.3% known as HNA-2-positive) and was absent on neutrophil surface of 4 donors (2.7% known as HNA-2-negative). Atypical expression of HNA-2 (three peaks) was observed in 9 (6%) donors. HNA-2 expression was considered negative if CD177+ cells were less than 5% [20]. The range of CD177 expression was 0.03-95.3 (Mean ± SD: 58.2 ± 21.7). The HNA-2 expression showed no significant difference among different age groups (*p* = 0.3) (Table 5).

A summary of the distribution of HNA allele frequencies among different ethnicities in the study cohort is depicted in Table 6.

## 8. Comparison of HNA Allele Frequencies among Iranian and Other Populations

The HNA-1, HNA-4, and HNA-5 genotype frequencies of Iranian blood donors showed no significant difference from the genotype reported for British and German populations (Table 7, *p* > 0.05). However, the HNA-1 genotype frequencies in the studied cohort were significantly different from the Hong Kong, Chinese sample form Guangzhou, Danish, and Zambians reports (*p* < 0.05). Similarly, for HNA-3 antigen, the genotype frequency in our cohort was different from the reported frequencies from Hong Kong, Guangzhou, British, German, Danish, and Zambians (all *p* < 0.05).

## 9. Discussion

In the last years, multiple attempts have been conducted for the detection of HNA genotyping and allele frequencies all over the world. Considering the importance of HNA allo and autoimmunizations in the mechanism of diseases such as TRALI, NAIN, AIN, and graft rejection, the data on HNA allele frequencies in a population may help to draw a picture for possible allo or auto immunization rate that can support therefore the prevention strategies.

The allele frequencies of HNAs have been widely studied all over the world. To date, HNA allele frequencies have been studied for some populations including Chinese [21], German [11], Thai [10], British [22], and Japanese [7]. Studies revealed significant variation in HNA allele frequencies among different populations. The current study is aimed at analyzing the HNA alleles in Iranian population.

Our analysis showed a similar frequencies for HNA-1, HNA-4, and HNA-5 alleles with the previous reports from German and British populations [22]. However, these frequencies were significantly different from the Chinese populations [9, 21]. In contrast to Japanese and Chinese reports and similar to Caucasian reports, in the current cohort, FCGR3B∗02 was the most frequently detected HNA-1 allele [7, 9, 11, 13, 15, 21–23].

Recently, the molecular mechanism regulating the HNA2 expression on the neutrophil surface has been partially identified [24]. Genetic mutations and gene polymorphisms responsible for HNA-2 expression deficiency have been investigated in some studies. A nonsense polymorphism has been reported to be associated with the absence of CD177 on the neutrophil surface [25]. Besides, it has been revealed that frameshift mutations in CD177 gene [26] and c.787A > T polymorphism in CD177 in combination with c.997delG or alone are involved in the absence of CD177 antigen on the neutrophil surface [27]. Antibodies against HNA-2 antigen participate in the mechanism of autoimmune neutropenia, allo-immune neutropenia, TRALI, and graft rejection [28–31]. Therefore, the analysis of HNA-2 expression is important to predict the immunization rate against this antigen. In the current study, HNA-2 expression was identified in 146 (97.3%) donors. Similar studies have shown presence of HNA2 on neutrophil surface of 98.7% population in Japanese [7], 97% in Brazilians [32], 86% in Koreans [33], and 99.55% in Thai blood donors [19].

HNA-2 null phenotype (neutrophils with no detectable CD177) has been observed in 3-5% of normal individuals [24]. The findings of the current study indicated a high HNA-2 expression on the neutrophil surface of the majority of the Iranian population. Furthermore, our findings revealed atypical expression of HNA-2 (two distinct positive cell populations) in 6% of donors. Previous studies have reported atypical CD177 expression in 8.5-11.5% of the normal individuals with HNA-2 atypical expression [34, 35].

A previous study on HNA-3 alleles showed a frequency of 0.74 and 0.26 for SLC44A2∗1 and SLC44A2∗2 alleles, respectively [17]. Similarly, the current analysis showed SLC44A2∗1 as the dominant allele with a 0.63 frequency. Interestingly, this frequency is significantly different from other reports in the other populations [7, 9, 11]. The results of HNA-5 typing in 190 Azerbaijani blood donors showed 0.51 and 0.49 frequencies for ITGAL∗1 and ITGAL∗2, respectively [16]. Similarly, our findings indicated ITGAL∗1 as a common allele. Similar results have been obtained from Malay, Chinese, and Indian populations [8].

Iranian population contains high ethnic diversity. The majority of the Iranian population are Persians. Persians and Azerbaijanis make up 70 percent of the population in Iran [36]. The cohort size has limited our analysis on the frequency of different HNA alleles among different ethnic groups in Iran. Hence, further studies with a larger sample size are required to draw a total picture of the distribution of the HNA allele and genotype among the different Iranian ethnic groups.

The similarity of some HNA allele frequencies between Iranian and some well-studied populations reported in the other studies may help to predict an immunization figure for those antigens in the Iranian population. Following the current data, a precise immunization rate against HNA alleles required a complementary study on antibody detection.

## Figures and Tables

**Table 1:** The characteristics of study participants. The participants were categorized based on age, gender, and ethnicity.

Gender	Ages			Ethnicity		
	Frequency		Percent	Frequency		Percent
Female	17-35	6	33.3	Persians	13	72.2
	36-50	7	38.9	Azerbaijanis	3	16.7
	>50	5	27.8	Gilaks and Mazandaranis	2	11.1
	Total	18	100	Total	18	100

Male	17-35	34	25.8	Persians	82	62.1
	36-50	65	49.2	Azerbaijanis	34	25.8
	>50	33	25	Kurds	5	3.8
	Total	132	100	Gilaks and Mazandaranis	4	3
				Lurs	6	4.5
				Arabs	1	0.8
				Total	132	100

**Table 2:** List of primer sequences for HNA genotyping using PCR-SSP.

HNA antigens	Primer sequence 5 → 3	Amplification size (based Paris)	References
HNA-1a	HNA1a F CAATGGTACAGGGTGCTC	115	[13]
	HNA1a R CCTGGCTTGAGATGAGGT		
HNA-1b	HNA1b F CCTCAATGGTACAGCGTGCTT	159	[13]
	HNA1b R ACTGTCGTTGACTGTGTCAG		
HNA-1c	HNA1c F CCTCAATGGTACAGCGTGCTT	160	[13]
	HNA1c R CACTGTCGTTGACTGTGTCAT		
HNA-3a	HNA3a F AGT GGC TGA GGT GCT TCG	291	^∗^
	HNA3a R GTG CGC CAA TAT CCT CAC TTG		
HNA-3b	HNA3b F GAGTGGCTGAGGTGCTTCA	292	^∗^
	HNA3b R GTG CGC CAA TAT CCT CAC TTG		
HNA-4a	HNA4a F TCA TGC GAG CCC ATC CG	248	^∗^
	HNA4a R ACA AGG AGG TCT GAC GGT G		
HNA-4b	HNA4b F CTC ATG CGA GCC CAT CCA	249	^∗^
	HNA4b R ACA AGG AGG TCT GAC GGT G		
HNA-5a	HNA5a F TTCTGATATTCCCCACCCTG	82	[13]
	HNA5a R CAGTTAGACGCAGGGCTC		
HNA-5b	HNA5b F TTCTGATATTCCCCACCCTG	84	[13]
	HNA5b R AGCAGTTAGACGCAGGGCTG		
Internal control	HGHF CAG TGC CTT CCCAACCATTCCCTTA	495	[13]
	HGHR ATC CAC TCA CGG ATT TCT GTT GTG TTT C		

HNA: human neutrophil antigen; F: forward; R: reverse; HGH: human growth hormone. ^∗^The primer sequences were obtained from the ISBT reference granulocyte laboratory in Giessen, Germany.

**Table 3:** Allele frequency of HNAs in Iranian blood donors.

HNA antigens	Alleles	Number of alleles	Frequency
HNA-1a	FCGR3B^∗^1	100	0.34
HNA-1b	FCGR3B^∗^2	189	0.63
HNA-1c	FCGR3B^∗^3	9	0.03
Total number of alleles	300^1^		
HNA-3a	SLC44A2^∗^1	189	0.63
HNA-3b	SLC44A2^∗^2	111	0.37
Total number of alleles	300		
HNA-4a	ITGAM^∗^1	256	0.85
HNA-4b	ITGAM^∗^2	44	0.15
Total number of alleles	300		
HNA-5a	ITGAL^∗^1	215	0.72
HNA-5b	ITGAL^∗^2	85	0.28
Total number of alleles	300		

^1^One sample with HNA1 null.

**Table 4:** The distribution of HNA genotype frequencies. HNA genotype frequency distribution in the Iranian population tested for the deviation of Hardy–Weinberg equilibrium.

Genotype frequency	Observed		Expected		Hardy–Weinberg analysis	
	Number	%	Number	%	*X* ^2^	*p* value
HNA1aa	21	14	16.7	11.3	861	0.125
HNA1bb	65	43	59.9	40.2		
HNA1ab	55	37	63.4	42.6		
HNA1ac	3	2	3.02	2.00		
HNA1bc	4	3	5.70	3.8		
HNA1cc	1	1	0.13	0.1		
HNA3aa	58	38.7	57.7	38.5	0.448	0.799
HNA3bb	19	12.7	21.8	14.5		
HNA3ab	73	48.6	70.5	47		
HNA4aa	110	73.3	108	72	0.601	0.740
HNA4bb	4	2.7	3	2		
HNA4ab	36	24	39	26		
HNA5aa	77	51.3	78.2	52	0.179	0.915
HNA5bb	12	8	10.7	7		
HNA5ab	61	40.7	61.1	41		

HNAs: human neutrophil antigens; *χ*2: chi-squared test value. No significant deviation from Hardy–Weinberg equilibrium was observed (*p* > 0.05).

**Table 5:** Distribution of the HNA-2 among Iranian blood donors based on sex and age.

Gender			HNA-2				Total
			More than 60%	50%-60%	5%-50%	Less than 5%	
Female	Age	17-35	5	1	0	—	6
		36-50	4	1	2	—	7
		>50	3	0	2	—	5
		Total	12	2	4	0	18

Male	Age	17-35	18	6	10	0	34
		36-50	35	8	19	3	65
		>50	16	5	11	1	33
		Total	69	19	40	4	132

**Table 6:** The distribution of HNA allele frequencies among different ethnicities in the study cohort.

		Ethnicity					
		Persians	Azerbaijanis	Kurds	Gilaks/Mazandaranis	Lurs	Arabs
HNA1	aa	15	5	1	0	0	0
	bb	38	22	2	0	3	0
	cc	1	0	0	0	0	0
	ab	37	8	2	5	3	0
	ac	1	0	0	1	0	1
	bc	2	2	0	0	0	0
	a-b-c	1	0	0	0	0	0
	Total	95	37	5	6	6	1
HNA2	>60%	53	17	3	4	3	1
	50%-60%	12	6	1	1	1	0
	5%-50%	28	13	1	1	1	0
	<5%	2	1	0	0	1	0
	Total	95	37	5	6	6	1
HNA3	aa	38	12	3	2	2	1
	bb	11	6	1	0	1	0
	ab	46	19	1	4	3	0
	Total	95	37	5	6	6	1
HNA4	aa	70	30	2	4	3	1
	bb	2	1	0	0	1	0
	ab	23	6	3	2	2	0
	Total	95	37	5	6	6	1
HNA5	aa	45	22	0	5	5	0
	bb	8	4	0	0	0	0
	ab	42	11	5	1	1	1
	Total	95	37	5	6	6	1

**Table 7:** Frequencies of HNA genotypes among different populations. NS: not shown.

Genotype frequency of HNA among Iranians, Chinese, and Caucasians.													
		Hong Kong, China		Guangzhou		British		German		Danish		Zambians	
		(Tam et al. [9])		Han (Xia et al. [21])		(Cardoso et al. [22])		(Hauck et al. [11])		(Nielsen et al. [15])		(Nielsen et al. [15])	
Genotype	Current study	Homogeneity test		Homogeneity test		Homogeneity test		Homogeneity test		Homogeneity test		Homogeneity test	
HNA1aa	14	45.7	*x* ^2^ = 101.9	42.6	*x* ^2^ = 137.817	7.9	*x* ^2^ = 13.63	15.1	*x* ^2^ = 9.2	13	*x* ^2^ = 14.2	19	*x* ^2^ = 69.53
HNA1bb	43	9.3	df = 6	9.1	df = 6	42.9	df = 7	36.1	df = 6	42	df = 7	16	df = 7
HNA1cc	1	0	*p* ≤ 0.01	0	*p* ≤ 0.01	0	*p* = 0.058	0	*p* = 157	0	*p* = 0.047	12	*p* ≤ 0.01
HNA1ab	37	44.3		48.3		46.4		47.9		39		25	
HNA1ac	2	0		0		0		0		2		15	
HNA1bc	3	0		0		0.7		0		0		10	
HNA1abc	0	0		0		2.1		NS		4		3	
HNA1a-b-c	1	0.7		0		0		0.9		0		1	
HNA3aa	38.7	51.4	*x* ^2^ = 6.5	53.3	*x* ^2^ = 14.2	56.4	*x* ^2^ = 14.6	55.5	*x* ^2^ = 8.1	67	*x* ^2^ = 38.2	95	*x* ^2^ = 128.2
HNA3ab	48.6	39.3	df = 2	41	df = 2	40.7	df = 2	37.8	df = 2	29	df = 2	5	df = 2
HNA3bb	12.7	9.3	*p* = 0.038	5.6	*p* ≤ 0.01	2.9	*p* ≤ 0.01	6.7	*p* = 0.017	4	*p* ≤ 0.01	0	*p* ≤ 0.01
HNA4aa	73.3	99	*x* ^2^ = 76.3	99.2	*x* ^2^ = 120.8	78.6	*x* ^2^ = 1.08	83.2	*x* ^2^ = 3.7	78	*x* ^2^ = 1.2	81	*x* ^2^ = 2.5
HNA4ab	24	1	df = 2	0.8	df = 2	19.3	df = 2	15.1	df = 2	21	df = 2	17	df = 2
HNA4bb	2.7	0	*p* ≤ 0.01	0	*p* ≤ 0.01	2.1	*p* = 0.58	1.7	*p* = 0.15	1	*p* = 0.53	2	*p* = 0.27
HNA5aa	51.3	71.7	*x* ^2^ = 24.7	73.4	*x* ^2^ = 29.8	54.3	*x* ^2^ = 0.27	55.5	*x* ^2^ = 0.83	53.0	*x* ^2^ = 0.9	22	*x* ^2^ = 34.57
HNA5ab	40.7	27	df = 2	24.3	df = 2	38.6	df = 2	35.3	df = 2	39.0	df = 2	56	df = 2
HNA5bb	8	1.3	*p* ≤ 0.01	2.4	*p* ≤ 0.01	7.1	*p* = 0.87	9.2	*p* = 0.65	8	*p* = 0.95	22	*p* ≤ 0.01

## Data Availability

All results were included in the manuscript. Data are available on request.
